# Supplementary material for: Best practices for implementing biosafety inspections in a clinical laboratory: Evidence from a multi-site experimental study
Source: PLoS One. 2023 Oct 13;18(10):e0292940. doi: 10.1371/journal.pone.0292940 (PMC10575490; doi:10.1371/journal.pone.0292940)
Supplement: S1 File — (DOCX) [file pone.0292940.s001.docx]

Inclusivity in global research

PLOS’ policy on inclusivity in global research aims to improve transparency in the reporting of research performed outside of researchers’ own country or community and ensures that PLOS publications reporting global research adhere to high standards for research ethics and authorship. Authors of relevant research articles may be asked to complete the questionnaire below, which outlines ethical, cultural, and scientific considerations specific to inclusivity in global research. This questionnaire may be requested when researchers have travelled to a different country to conduct research, if research uses samples collected in another country, research with Indigenous populations or their lands, or if research is on cultural artefacts. Researchers travelling to another country solely to use laboratory equipment will not normally be required to complete the questionnaire. However, the questionnaire can be requested at the journal’s discretion for any submission – if you have been requested to complete this questionnaire by the PLOS journal you submitted to, please do so.

Please complete the questionnaire below and include this as a Supporting Information file with your manuscript. Note that if your paper is accepted for publication, this checklist will be published with your article in the supporting information files. Please ensure that you reference the checklist in the main body of your manuscript. We suggest adding a subsection ‘Inclusivity in global research’ to your Methods section and adding the following sentence: “Additional information regarding the ethical, cultural, and scientific considerations specific to inclusivity in global research is included in the Supporting Information (SX Checklist)”

The questions have been designed to be applicable to a wide range of study types, and there are subsections for both human subjects research and non-human subjects research. If any of the questions are not relevant to your research please mark them as “N/A” as appropriate.

**Ethical considerations, permits and authorship**

*This section is applicable to all research types.*

Provide details as to who granted permissions and/or consent for the study to take place in the Methods section of your manuscript. This should include the names of **all** ethics boards, governmental organizations, community leaders or other bodies that provided approval for the study. If individuals provided approval refer to these people by their role or title but do not list their name(s).

Reported on page number: 7

If there were any deviations from the study protocol after approval was obtained please provide details of these changes in the Methods section of your manuscript.
Did this study involve local collaborators that are residents of the country where the research was conducted or members of the community studied? If you do not have any authors from said communities, please provide an explanation for this below.

Reported on page number: there is no deviation

Everyone listed as an author should meet PLOS’ criteria for authorship and all individuals who meet these criteria should be included in the author byline, rather than the acknowledgements. Authorship criteria is based on the International Committee of Medical Journal Editors (ICMJE) Uniform Requirements for Manuscripts Submitted to Biomedical Journals - for further information please see here: <https://journals.plos.org/plosone/s/authorship>.

Yes

**Human subjects research (e.g. health research, medical research, cross-cultural psychology)**

Did you obtain written informed consent from a representative of the local community or region before the research took place? How did you establish who speaks for the community? Details of written informed consent obtained from study participants should be reported separately in the Methods section of your manuscript.

Before conducting our research, we engaged in extensive communication with the clinical lab workers and obtained their written informed consent. These clinical lab workers completed a consent form prior to filling out the questionnaire, which included information about the purpose of the study, the procedures involved, and the confidentiality of their responses. We consider these clinical lab workers to be representatives of the community associated with the clinical laboratory, and their consent was obtained prior to the commencement of the research. Details of written informed consent obtained from study participants have been provided on page 7.

How did members of the local community provide input on the aims of the research investigation, its methodology, and its anticipated outcome(s)?

During the initial phase of the study design, we conducted a focus group discussion involving 3 directors and 5 clinical laboratory workers. This session provided an opportunity for members of the local community to provide input and contribute to the aims of the research investigation, its methodology, and its anticipated outcomes.

The directors and laboratory workers shared their perspectives, opinions, and suggestions, which helped shape various aspects of the research, including its objectives and focus. Their valuable insights and feedback were taken into consideration during the development of the research design, ensuring that the study aligns with the interests and needs of the local community.

When engaging with the local community, how did you ensure that the informed consent documents and other materials could be understood by local stakeholders?

When engaging with the local community, we took measures to ensure that the informed consent documents and other materials could be understood by local stakeholders. This included the use of clear and concise language in the documents, avoiding technical jargon. Additionally, we sought feedback from a representative sample of the local community to assess their understanding of the materials and made necessary revisions based on their input. This iterative process helped to ensure that the informed consent documents and other materials were accessible and comprehensible to the local stakeholders.

Will the findings of the research be made available in an understandable format to stakeholders in the community where the study was conducted (e.g. via a presentation, summary report, copies of publications, etc.)? Please provide details of how this will be achieved.

We took steps to ensure that the findings of the research were effectively communicated to stakeholders in the community where the study was conducted. A summary report was prepared to disseminate the research results to the participating laboratory directors. These directors, in turn, shared the findings with the clinical lab workers in their respective laboratories. The summary report was carefully designed using clear language and visual aids to facilitate understanding among the recipients.

Additionally, we organized meetings with the laboratory directors to provide them with the complete research report, allowing them to gain a comprehensive understanding of the study. This facilitated their ability to convey detailed information to the lab workers in their respective clinical laboratories.

Our objective was to promote effective communication and understanding of the research findings among the clinical lab workers. By providing both the summary report and the complete research report to the laboratory directors, who then shared the information with their staff, we aimed to enhance accessibility and comprehensibility of the research

**Non-human subjects research using specimens/ animals collected as part of the study, or those housed in archival collections. Examples include archaeology, paleontology, botany and zoology.**

Did the permission you obtained from a local authority to perform the study include an agreement on access to outputs and benefit sharing? This may include procedures to enable fair distribution of the benefits and resources arising from the research performed. Please include any details of Prior Informed Consent and Benefit Sharing Agreements obtained. These may be required by field-specific regulations, for example the Convention on Biological Diversity (CBD) and the associated Nagoya Protocol.

N/A

If the material used in your study was imported, please A) provide the year it was imported and B) indicate whether permits were obtained to import/export the materials used, C) provide details of any permits obtained. If this information is not available, please indicate this.

N/A

If you used archival specimens, please state how the material used in your study was acquired by the institute it is held in and provide details of any permits obtained for the original excavations/ sample collection. If this information is not available, please indicate this.

N/A

How was the potential cultural significance of the materials collected in your study to local communities considered in your research design? Were Indigenous peoples and/or local researchers and institutions involved with archaeological excavations / collection of specimens? If so, please provide a description of their involvement.

N/A

If your manuscript includes photographs of human remains please indicate whether authors obtained permission from descendants or affiliated cultural communities to do so.

N/A
