# Supplementary material for: Best practices for implementing biosafety inspections in a clinical laboratory: Evidence from a multi-site experimental study
Source: PLoS One. 2023 Oct 13;18(10):e0292940. doi: 10.1371/journal.pone.0292940 (PMC10575490; doi:10.1371/journal.pone.0292940)
Supplement: S3 File — A. Probability calculation of a scenario–An example. B. Probability calculation for all scenarios. (ZIP) [file pone.0292940.s003.zip › S3A.docx]

**The probability analysis**

In our discrete choice experiment, the explainable utility $V_{ij}$ for clinical lab worker $i$ to choose alternative $j$ is

$V_{ij}=\beta_{1}{GROUP}_{ij}+\beta_{2}{SAFETY}_{ij}+\beta_{3}{EXTERNAL}_{ij}+\beta_{4}{MONTHLY}_{ij}+\beta_{5}{BEFORE}_{ij}+\beta_{6}{AFTER}_{ij}+\beta_{7}{RANDOM}_{ij}+\beta_{8}{EMAIL}_{ij}+\beta_{9}{SUPERVISOR}_{ij}+\beta_{10}{POST}_{ij}+\beta_{11}{DISCUSSION}_{ij}+\beta_{12}{RETRAIN}_{ij}+\beta_{13}{RECOGNITION}_{ij}$ (A1)

In a conditional logit model, individuals are assumed to be homogeneous. Therefore, the coefficient vector $\boldsymbol{\beta}$ in Equation (A1) is independent of the respondents, and the index $i$ in Equation (A1) can be dropped. In general, the probability of choosing alternative $j$ over alternative $k$ can be calculated as

$P_{j}={e^{V_{j}}}/{(e^{V_{j}}+e^{V_{k}})}$ (A2)

Since the choice in our study is binary (yes or no), Equation (A2) for calculating a probability can be further simplified as

$P_{yes}={e^{V_{yes}}}/{(1+e^{V_{yes}})}$ (A3)

Because effects coding was used in our study, the number of new variables created in effects coding was equivalent to the number of levels of the attribute being coded, minus one. For instance, there were four levels in the attribute of lab safety inspector. We created three new variables, such as GROUP, SAFETY, and EXTERNAL.

Next, we use the baseline intervention as an example to calculate its corresponding probability of compliance for this intervention. Note that in the baseline intervention, all attributes were set to their reference levels, and the coding for these reference levels was presented in Table 1. For example, for the attribute of lab safety inspector, “lab director” was defined as the reference level. Therefore, we coded GROUP, SAFETY, and EXTERNAL to be -1 to represent the lab director.

Using the coefficients of the regression results shown in Table 3, the explainable utility $V$ at the baseline intervention is equal to

$$V=-0.0671\times(-1)+0.2155\times(-1)-0.1205\times(-1)+0.2493\times(-1)-0.0087\times(-1)-0.3451\times(-1)+0.0766\times(-1)+0.1776\times(-1)+0.0914\times(-1)+0.0618\times(-1)+0.0435\times(-1)+0.3558\times(-1)+0.3231\times(-1)=-1.0532$$

Furthermore, plugging $V=-1.0532$ into $V_{yes}$ in Equation (A3) yields the probability of the baseline intervention as 25.86%.
