# Supplementary material for: Best practices for implementing biosafety inspections in a clinical laboratory: Evidence from a multi-site experimental study
Source: PLoS One. 2023 Oct 13;18(10):e0292940. doi: 10.1371/journal.pone.0292940 (PMC10575490; doi:10.1371/journal.pone.0292940)
Supplement: S1 Table — (DOCX) [file pone.0292940.s004.docx]

S4 Table. Regression results for various groups based on age

|  |  | <26 | | 26-35 | | >36 | |
| --- | --- | --- | --- | --- | --- | --- | --- |
| Attributes | Levels | Coefficients | Standard  error | Coefficients | Standard  error | Coefficients | Standard  error |
| Lab Safety Inspector | By a group leader | -0.4125** | 0.1560 | 0.0601 | 0.0851 | -0.1180 | 0.1319 |
|  | By a safety committee member | 0.3163* | 0.1490 | 0.2496** | 0.0892 | 0.0888 | 0.1252 |
|  | By an external expert | -0.0135 | 0.1486 | -0.2111* | 0.0832 | 0.0105 | 0.1311 |
| Inspection Frequency | Monthly | 0.3669* | 0.1542 | 0.2620** | 0.0877 | 0.1800 | 0.1365 |
|  | Before an audit | -0.4912** | 0.1591 | 0.1415 | 0.0893 | 0.0713 | 0.1356 |
|  | After a safety incident | -0.2631* | 0.1394 | -0. 4310*** | 0.0864 | -0.2554* | 0.1250 |
| Inspection Timing | Random day and time | 0.0463 | 0.0723 | 0.0345 | 0.0425 | 0.2166** | 0.0663 |
| Communication of Outcome | By an individual email | 0.2413* | 0.1402 | 0.1531* | 0.0911 | 0.1991 | 0.1247 |
|  | By a supervisor, given verbally | 0.0286 | 0.1398 | 0.0846 | 0.0829 | 0.1477 | 0.1212 |
|  | Outcome posted publicly | 0.0873 | 0.1395 | 0.0909 | 0.0823 | -0.0263 | 0.1278 |
| Reward / Punishment | Meet a supervisor if unsatisfactory | -0.1506 | 0.1465 | 0.1054 | 0.0863 | 0.0485 | 0.1295 |
|  | Receive retraining if unsatisfactory | 0.4007** | 0.1503 | 0.4146*** | 0.0912 | 0.2095 | 0.1286 |
|  | Receive recognition if satisfactory | 0.5095*** | 0.1458 | 0.2960*** | 0.0843 | 0.2981* | 0.1358 |
| ***p<0.001, **p<0.010, *p<0.100 | | | | | |  | |
