# Supplementary material for: Best practices for implementing biosafety inspections in a clinical laboratory: Evidence from a multi-site experimental study
Source: PLoS One. 2023 Oct 13;18(10):e0292940. doi: 10.1371/journal.pone.0292940 (PMC10575490; doi:10.1371/journal.pone.0292940)
Supplement: S2 Table — (DOCX) [file pone.0292940.s005.docx]

S5 Table. Regression results for various groups based on gender

|  |  | Male | | Female | |
| --- | --- | --- | --- | --- | --- |
| Attributes | Levels | Coefficients | Standard  error | Coefficients | Standard  error |
| Lab Safety Inspector | By a group leader | -0.0929 | 0.1211 | -0.0615 | 0.0743 |
|  | By a safety committee member | 0.4409*** | 0.1236 | 0.1316* | 0.0744 |
|  | By an external expert | -0.3276** | 0.1166 | -0.0406 | 0.0746 |
| Inspection Frequency | Monthly | 0.3386** | 0.1228 | 0.2136** | 0.0764 |
|  | Before an audit | 0.0476 | 0.1251 | -0.0291 | 0.0777 |
|  | After a safety incident | -0.3329** | 0.1178 | -0.3485*** | 0.0733 |
| Inspection Timing | Random day and time | 0.0818 | 0.0582 | 0.0764* | 0.0373 |
| Communication of Outcome | By an individual email | 0.1209 | 0.1222 | 0.1978** | 0.0755 |
|  | By a supervisor, given verbally | 0.1149 | 0.1142 | 0.0824 | 0.0709 |
|  | Outcome posted publicly | -0.0097 | 0.1127 | 0.0949 | 0.0726 |
| Reward / Punishment | Meet a supervisor if unsatisfactory | 0.0628 | 0.1200 | 0.0340 | 0.0749 |
|  | Receive retraining if unsatisfactory | 0.1913 | 0.1241 | 0.4214*** | 0.0780 |
|  | Receive recognition if satisfactory | -0.3107** | 0.1187 | -0.3337*** | 0.0749 |
| ***p<0.001, **p<0.010, *p<0.100 | | | | | |
