# Supplementary material for: Best practices for implementing biosafety inspections in a clinical laboratory: Evidence from a multi-site experimental study
Source: PLoS One. 2023 Oct 13;18(10):e0292940. doi: 10.1371/journal.pone.0292940 (PMC10575490; doi:10.1371/journal.pone.0292940)
Supplement: S3 Table — (DOCX) [file pone.0292940.s006.docx]

S6 Table. Regression results for various groups based on education

|  |  | Junior college or lower | | Undergraduate | | Postgraduate or above | |
| --- | --- | --- | --- | --- | --- | --- | --- |
| Attributes | Levels | Coefficients | Standard  error | Coefficients | Standard  error | Coefficients | Standard  error |
| Lab Safety Inspector | By a group leader | -0.3310* | 0.1370 | 0.0436 | 0.0897 | -0.0583 | 0.1247 |
|  | By a safety committee member | 0.2305* | 0.1292 | 0.2660** | 0.0911 | 0.1243 | 0.1248 |
|  | By an external expert | 0.0058 | 0.1336 | -0.2073* | 0.0885 | -0.0950 | 0.1222 |
| Inspection Frequency | Monthly | 0.2702* | 0.1369 | 0.2833** | 0.0911 | 0.1776 | 0.1273 |
|  | Before an audit | -0.2124 | 0.1390 | 0.0279 | 0.0944 | 0.0770 | 0.1274 |
|  | After a safety incident | -0.3596** | 0.1273 | -0.3563*** | 0.0878 | -0.3315* | 0.1292 |
| Inspection Timing | Random day and time | 0.0189 | 0.0656 | 0.0686 | 0.0445 | 0.1548* | 0.0615 |
| Communication of Outcome | By an individual email | 0.2265* | 0.1280 | 0.0630 | 0.0929 | 0.3488** | 0.1278 |
|  | By a supervisor, given verbally | 0.0353 | 0.1245 | 0.1512* | 0.0863 | 0.0431 | 0.1166 |
|  | Outcome posted publicly | 0.1361 | 0.1264 | 0.0753 | 0.0858 | -0.0412 | 0.1206 |
| Reward / Punishment | Meet a supervisor if unsatisfactory | -0.0307 | 0.1317 | 0.0298 | 0.0905 | 0.1685 | 0.1270 |
|  | Receive retraining if unsatisfactory | 0.3034* | 0.1347 | 0.3630*** | 0.0941 | 0.3938** | 0.1295 |
|  | Receive recognition if satisfactory | 0.4536** | 0.1333 | 0.3143*** | 0.0894 | 0.2449* | 0.1231 |
| ***p<0.001, **p<0.010, *p<0.100 | | | | | |  | |
