# Supplementary material for: Best practices for implementing biosafety inspections in a clinical laboratory: Evidence from a multi-site experimental study
Source: PLoS One. 2023 Oct 13;18(10):e0292940. doi: 10.1371/journal.pone.0292940 (PMC10575490; doi:10.1371/journal.pone.0292940)
Supplement: S4 Table — (DOCX) [file pone.0292940.s007.docx]

S7 Table. Regression results for various groups based on years of work experience

|  |  | <1 year | | 1-5 years | | 6-10 years | | >10 years | |
| --- | --- | --- | --- | --- | --- | --- | --- | --- | --- |
| Attributes | Levels | Coefficients | Standard  error | Coefficients | Standard  error | Coefficients | Standard  error | Coefficients | Standard  error |
| Lab Safety Inspector | By a group leader | -0.3955* | 0.1635 | 0.0178 | 0.1227 | 0.0108 | 0.1260 | -0.0393 | 0.1142 |
|  | By a safety committee member | 0.2505 | 0.1580 | 0.2150* | 0.1284 | 0.3229* | 0.1301 | 0.1321 | 0.1115 |
|  | By an external expert | 0.0817 | 0.1597 | -0.1651 | 0.1215 | -0.2548* | 0.1233 | -0.0952 | 0.1145 |
| Inspection Frequency | Monthly | 0.3168* | 0.1625 | 0.3643** | 0.1278 | 0.2036 | 0.1294 | 0.1725 | 0.1180 |
|  | Before an audit | -0.3295* | 0.1687 | -0.1012 | 0.1284 | 0.2192* | 0.1319 | 0.0639 | 0.1186 |
|  | After a safety incident | -0.4176** | 0.1487 | -0.3125* | 0.1241 | -0.4491** | 0.1292 | -0.2435* | 0.1102 |
| Inspection Timing | Random day and time | 0.0238 | 0.0785 | 0.0214 | 0.0617 | 0.0794 | 0.0623 | 0.1598** | 0.0574 |
| Communication of Outcome | By an individual email | 0.1144 | 0.1499 | 0.2463* | 0.1320 | 0.1591 | 0.1341 | 0.1834 | 0.1117 |
|  | By a supervisor, given verbally | 0.0015 | 0.1503 | 0.0865 | 0.1209 | 0.0850 | 0.1204 | 0.1453 | 0.1074 |
|  | Outcome posted publicly | 0.1799 | 0.1512 | 0.0850 | 0.1205 | 0.0213 | 0.1205 | 0.0087 | 0.1111 |
| Reward / Punishment | Meet a supervisor if unsatisfactory | -0.1215 | 0.1566 | 0.0906 | 0.1261 | 0.1213 | 0.1277 | 0.0218 | 0.1144 |
|  | Receive retraining if unsatisfactory | 0.5082** | 0.1616 | 0.4278** | 0.1339 | 0.3198* | 0.1321 | 0.2582* | 0.1154 |
|  | Receive recognition if satisfactory | 0.3324* | 0.1574 | 0.2704* | 0.1210 | 0.4203** | 0.1251 | 0.3166** | 0.1168 |
| ***p<0.001, **p<0.010, *p<0.100 | | | | | |  | |  |  |
