# Supplementary material for: Best practices for implementing biosafety inspections in a clinical laboratory: Evidence from a multi-site experimental study
Source: PLoS One. 2023 Oct 13;18(10):e0292940. doi: 10.1371/journal.pone.0292940 (PMC10575490; doi:10.1371/journal.pone.0292940)
Supplement: S5 Table — (DOCX) [file pone.0292940.s008.docx]

S8 Table. Regression results for various groups based on employment type

|  |  | Permanent | | Contract | | Temporary | |
| --- | --- | --- | --- | --- | --- | --- | --- |
| Attributes | Levels | Coefficients | Standard  error | Coefficients | Standard  error | Coefficients | Standard  error |
| Lab Safety Inspector | By a group leader | -0.0743 | 0.1504 | -0.0259 | 0.0745 | -0.5631* | 0.2789 |
|  | By a safety committee member | 0.1279 | 0.1466 | 0.2350** | 0.0756 | 0.3518 | 0.2249 |
|  | By an external expert | -0.0860 | 0.1444 | -0.1747* | 0.0739 | 0.4990 | 0.4450 |
| Inspection Frequency | Monthly | 0.3997** | 0.1509 | 0.2032** | 0.0762 | 0.0487 | 0.3894 |
|  | Before an audit | 0.0950 | 0.1519 | 0.0141 | 0.0780 | -0.2899 | 0.3210 |
|  | After a safety incident | -0.3933* | 0.1534 | -0.3419*** | 0.0735 | -0.1233 | 0.2455 |
| Inspection Timing | Random day and time | 0.1788* | 0.0727 | 0.0593 | 0.0371 | 0.1745 | 0.1789 |
| Communication of Outcome | By an individual email | 0.1084 | 0.1485 | 0.2083** | 0.0770 | 0.1314 | 0.2264 |
|  | By a supervisor, given verbally | 0.1973 | 0.1417 | 0.0769 | 0.0714 | 0.2109 | 0.2355 |
|  | Outcome posted publicly | -0.0188 | 0.1413 | 0.0427 | 0.0720 | 0.6523* | 0.3656 |
| Reward / Punishment | Meet a supervisor if unsatisfactory | 0.1928 | 0.1520 | 0.0332 | 0.0754 | -0.2979 | 0.2528 |
|  | Receive retraining if unsatisfactory | 0.0061 | 0.1509 | 0.4401*** | 0.0786 | 0.5796* | 0.2525 |
|  | Receive recognition if satisfactory | 0.2315 | 0.1472 | 0.3077*** | 0.0740 | 1.1168* | 0.4343 |
| ***p<0.001, **p<0.010, *p<0.100 | | | | | |  | |
