# Supplementary material for: Best practices for implementing biosafety inspections in a clinical laboratory: Evidence from a multi-site experimental study
Source: PLoS One. 2023 Oct 13;18(10):e0292940. doi: 10.1371/journal.pone.0292940 (PMC10575490; doi:10.1371/journal.pone.0292940)
Supplement: S6 Table — (DOCX) [file pone.0292940.s009.docx]

S9 Table. Regression results for various groups based on lab size

|  |  | <50 | | 50-100 | | >100 | |
| --- | --- | --- | --- | --- | --- | --- | --- |
| Attributes | Levels | Coefficients | Standard  error | Coefficients | Standard  error | Coefficients | Standard  error |
| Lab Safety Inspector | By a group leader | -0.1982 | 0.1275 | -0.0456 | 0.0930 | -0.0007 | 0.1229 |
|  | By a safety committee member | 0.3260* | 0.1277 | 0.2028* | 0.0935 | 0.1602 | 0.1212 |
|  | By an external expert | -0.0423 | 0.1198 | -0.1512 | 0.0932 | -0.1365 | 0.1228 |
| Inspection Frequency | Monthly | 0.3318* | 0.1315 | 0.1673* | 0.0939 | 0.3359** | 0.1269 |
|  | Before an audit | 0.0292 | 0.1303 | 0.0128 | 0.0970 | -0.0729 | 0.1287 |
|  | After a safety incident | -0.2915* | 0.1188 | -0.4285*** | 0.0938 | -0.2948* | 0.1211 |
| Inspection Timing | Random day and time | 0.0768 | 0.0621 | 0.0330 | 0.0453 | 0.1536* | 0.0622 |
| Communication of Outcome | By an individual email | 0.2874* | 0.1264 | 0.1951* | 0.0959 | 0.0806 | 0.1256 |
|  | By a supervisor, given verbally | -0.0203 | 0.1164 | 0.1180 | 0.0892 | 0.1646 | 0.1172 |
|  | Outcome posted publicly | -0.0291 | 0.1191 | 0.0765 | 0.0885 | 0.0983 | 0.1200 |
| Reward / Punishment | Meet a supervisor if unsatisfactory | 0.0406 | 0.1212 | -0.0471 | 0.0959 | 0.1852 | 0.1241 |
|  | Receive retraining if unsatisfactory | 0.3951** | 0.1263 | 0.3602*** | 0.0992 | 0.3157* | 0.1255 |
|  | Receive recognition if satisfactory | 0.2899* | 0.1232 | 0.3359*** | 0.0920 | 0.3752** | 0.1245 |
| ***p<0.001, **p<0.010, *p<0.100 | | | | | |  | |
