# Supplementary material for: Best practices for implementing biosafety inspections in a clinical laboratory: Evidence from a multi-site experimental study
Source: PLoS One. 2023 Oct 13;18(10):e0292940. doi: 10.1371/journal.pone.0292940 (PMC10575490; doi:10.1371/journal.pone.0292940)

**INTRODUCTION TO THE SURVEY**

The purpose of this survey is to help us understand your workplace safety behavior in the clinical laboratory.

This survey has two parts. PART I asks about your basic information.

In PART II, you will be given two **hypothetic** scenarios, A and B. Each scenario is defined by five factors. The scenarios vary due to the description of the factors. You need to **imagine** that you are in the given scenarios, and then choose one of the scenarios in each question under which you are more motivated to comply with the lab’s safety guidelines.

**When you make the choice in the hypothetic question, we ask that you take it seriously, consider it carefully, and respond it as if you were actually facing the situation described in the question. Your answer will contribute to identifying the factors and improving the overall safety compliance in the lab.**

In total, there are 14 questions. The survey may take up to 10 minutes to complete. You can decide whether to participate in this study. Your decision will not have any influence on your performance evaluation. Any information obtained in this survey will remain confidential.

**PART I: YOUR BASIC INFORMATION**

**1.1** Your gender is

①Male ②Female

**1.2** Your age is

①$<$26 ②26-35 ③>36

**1.3** Your education level is

①Junior college or lower ②Undergraduate ③Postgraduate or above

**1.4** How many years have you worked in a laboratory?

①<1 ②1-5 ③6-10 ④>10

**1.5** Your employment type is

①Permanent ②Contract ③Temporary

**1.6** What is the size of the laboratory (No. of people) you worked in?

①$<$50 ②50-100 ③>100

**PART II**

**PLEASE READ**

- PART II has 8 choice questions. In each question, you are given two **hypothetic** scenarios, A and B. Please **imagine** you were in the given scenarios, and **choose one of the scenarios** in **each question** under which you are **more willing** to comply with the lab’s safety guidelines.
- Please place a ‘√’ in the chosen box.
- An example about a choice question is given next.

**
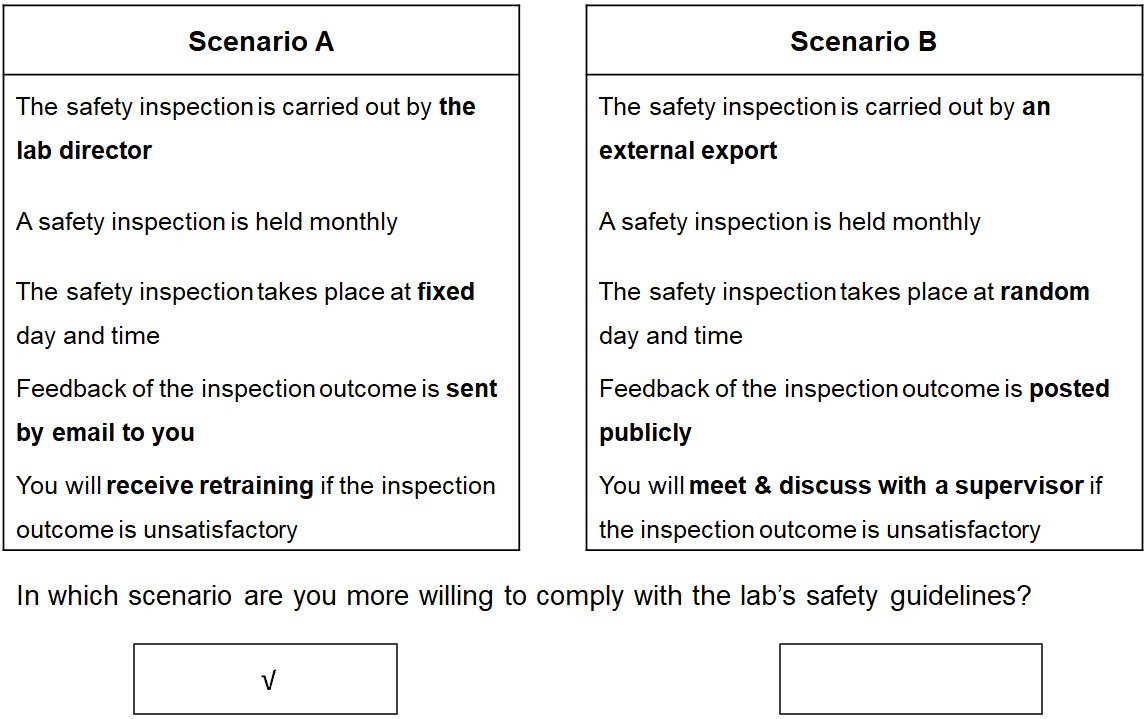
Example**

*When comparing with Scenarios A and B, the participant who answered this question was more willing to comply with the lab’s safety guidelines in Scenario A than in Scenario B.*

**START**

2.1


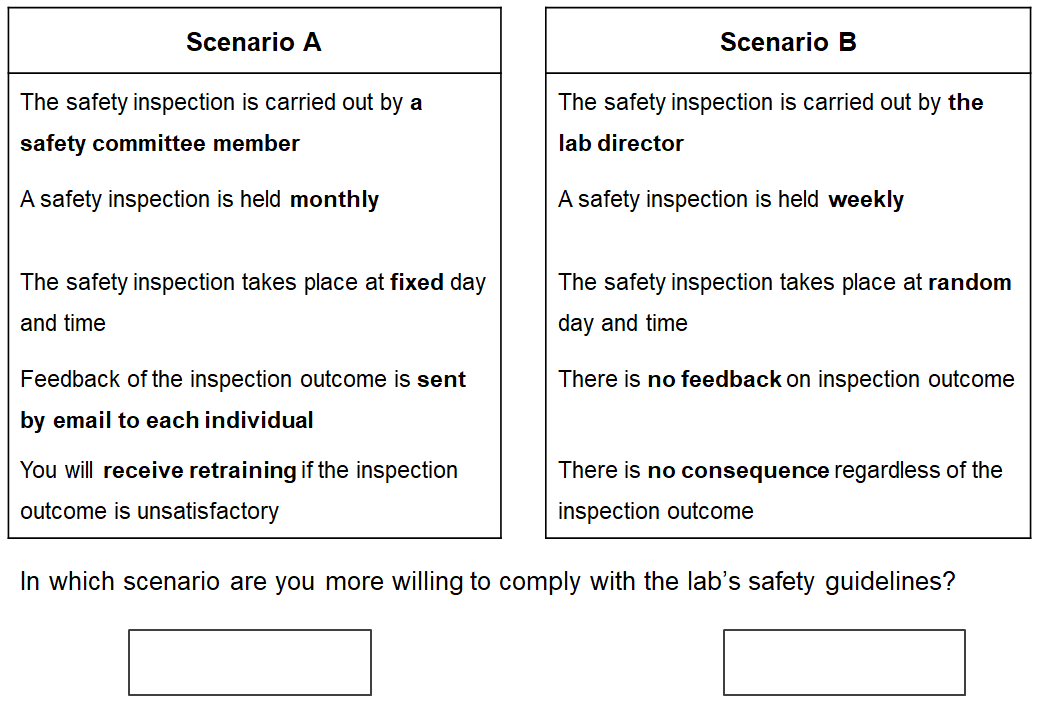


2.2


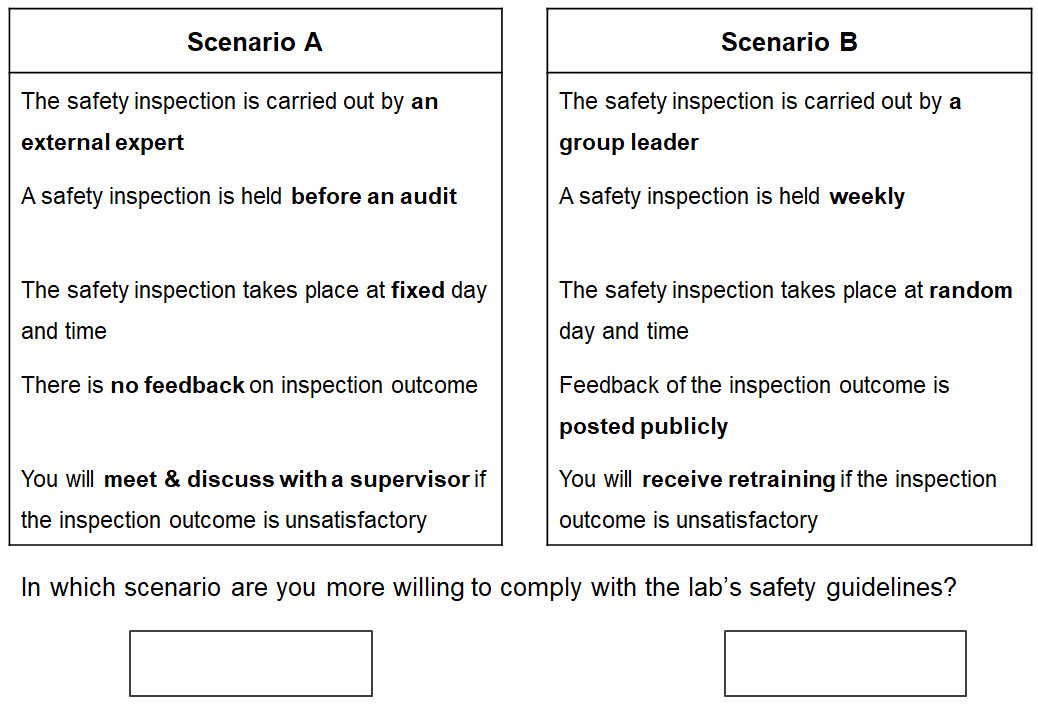


2.3


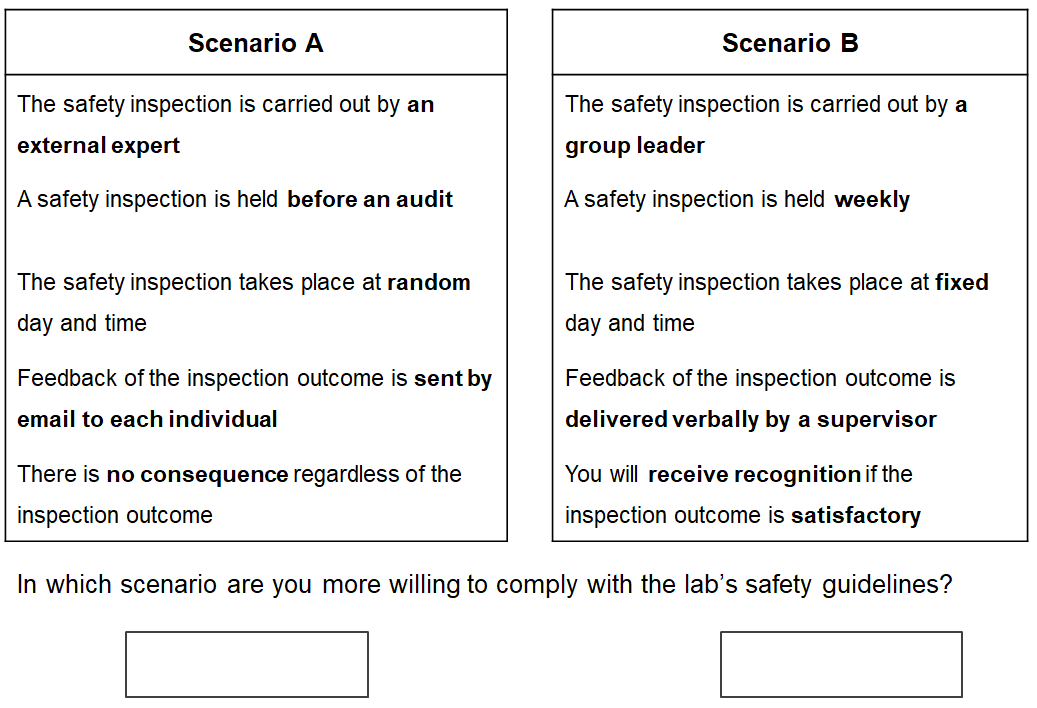


2.4


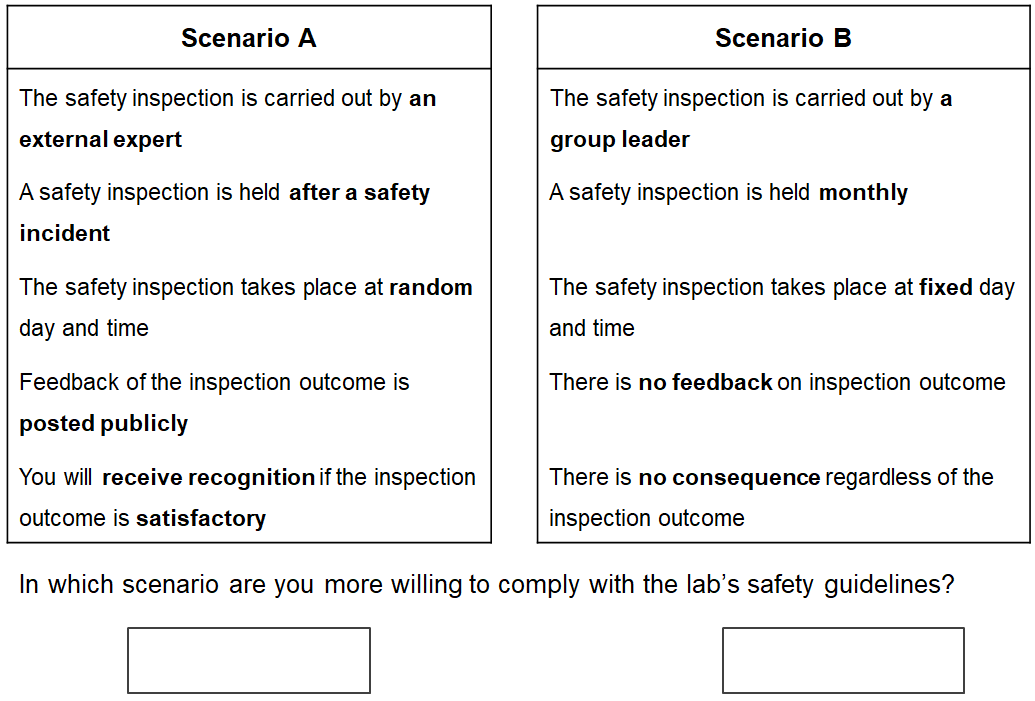


2.5


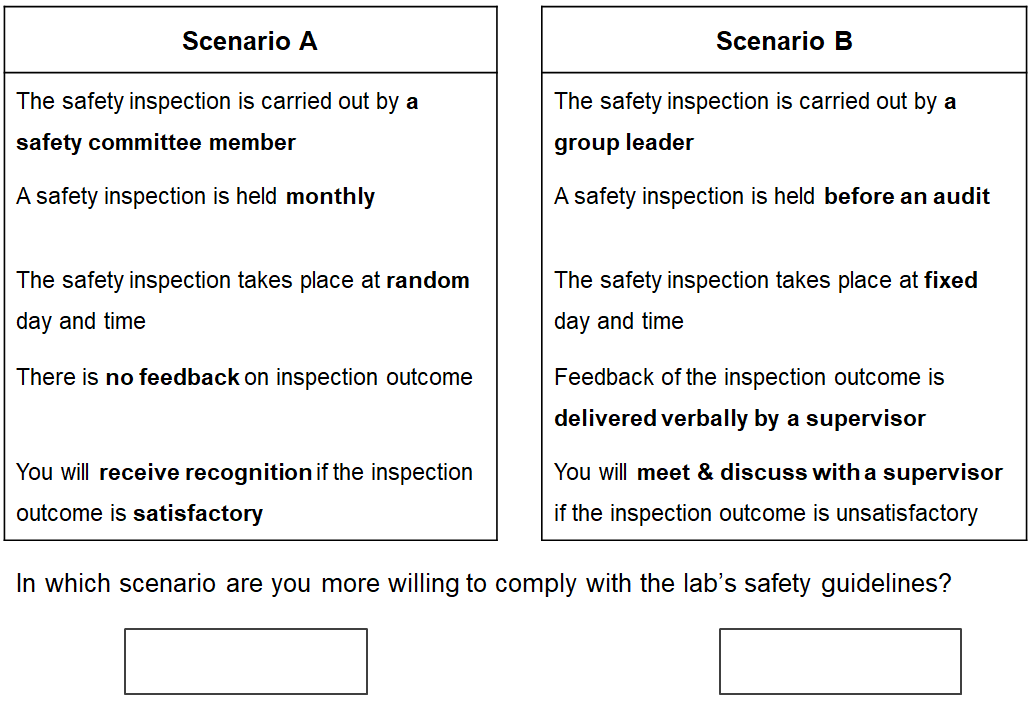


2.6


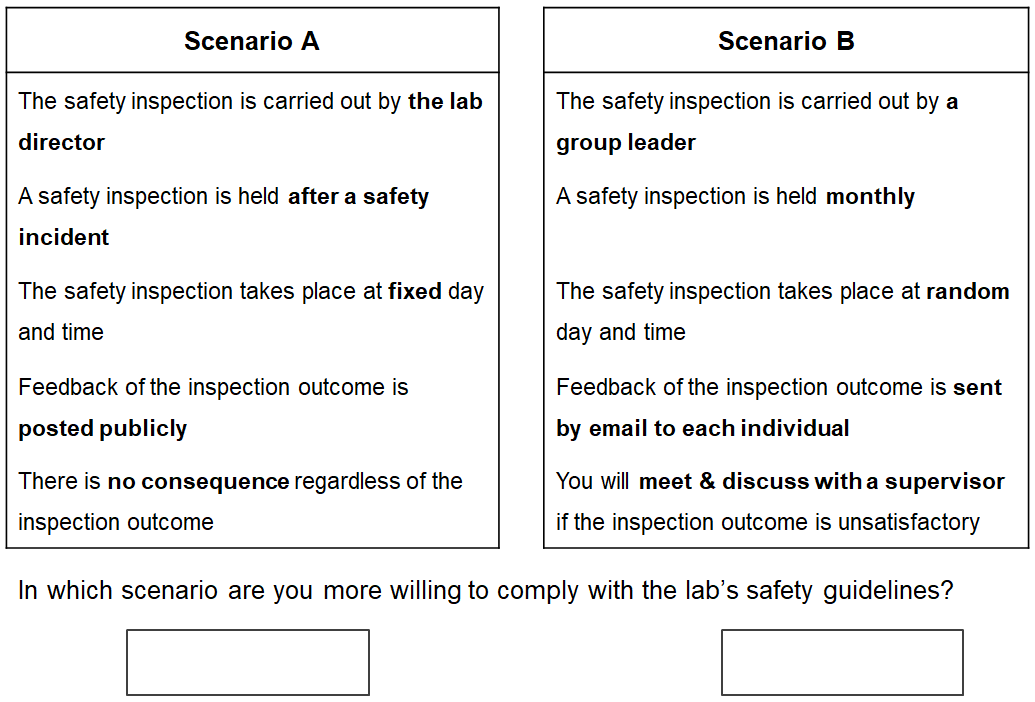


2.7


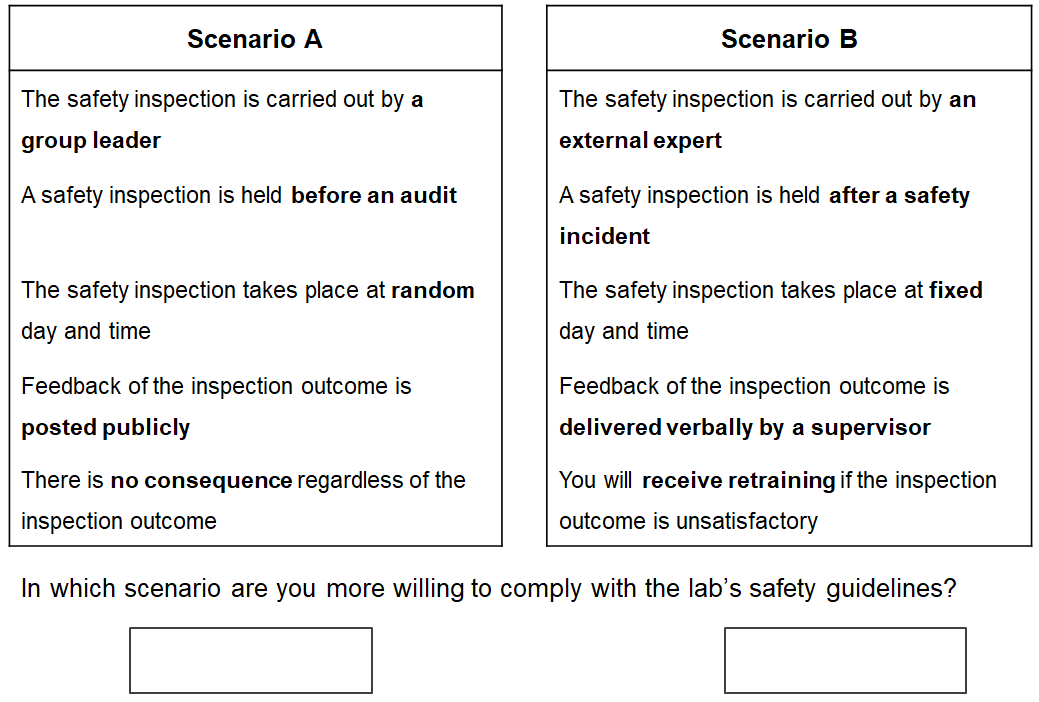


2.8


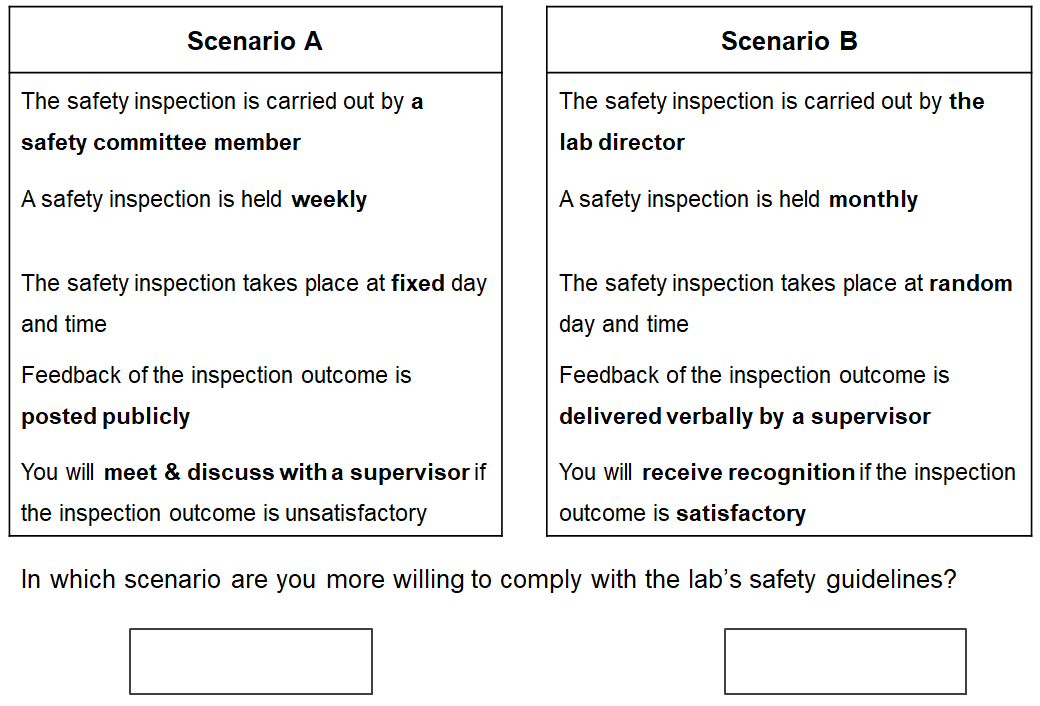

Supplement: S2 Questionnaire — (DOCX) [file pone.0292940.s011.docx]
