# Supplementary material for: Best practices for implementing biosafety inspections in a clinical laboratory: Evidence from a multi-site experimental study
Source: PLoS One. 2023 Oct 13;18(10):e0292940. doi: 10.1371/journal.pone.0292940 (PMC10575490; doi:10.1371/journal.pone.0292940)
Supplement: Attachment — Submitted filename: response letter.docx [file pone.0292940.s012.docx]

**Responses to the Reviewers’ Comments**

The authors would like to thank the review team for their thoughtful, thorough, and constructive feedback on our previous submission. We appreciate the opportunity to further strengthen our research. We have taken all reviewer comments to heart and tried our best to address all the raised concerns.

**Responses to the Academic editor**

1. Please ensure that your manuscript meets PLOS ONE's style requirements, including those for file naming. The PLOS ONE style templates can be found at https://journals.plos.org/plosone/s/file?id=wjVg/PLOSOne_formatting_sample_main_body.pdf and <https://journals.plos.org/plosone/s/file?id=ba62/PLOSOne_formatting_sample_title_authors_affiliations.pdf>

>>> Thank you for bringing this to our attention. We have carefully reviewed this manuscript to ensure that it adheres to PLOS ONE’s style requirements.

2. Please provide additional details regarding participant consent. In the ethics statement in the Methods and online submission information, please ensure that you have specified (1) whether consent was informed and (2) what type you obtained (for instance, written or verbal, and if verbal, how it was documented and witnessed). If your study included minors, state whether you obtained consent from parents or guardians. If the need for consent was waived by the ethics committee, please include this information. If you are reporting a retrospective study of medical records or archived samples, please ensure that you have discussed whether all data were fully anonymized before you accessed them and/or whether the IRB or ethics committee waived the requirement for informed consent. If patients provided informed written consent to have data from their medical records used in research, please include this information.

>>> The individuals who took part in this research were clinical laboratory workers. Their involvement in the study was completely voluntary, and prior to answering the questionnaire, each participant gave their informed consent by signing a written consent form.

The above information has been added to the Methods section of the manuscript on page 7.

3. Please include your ethics statement in the Methods section of your manuscript. In the Methods section of your revised manuscript, please include the full name of the institutional review board or ethics committee that approved the protocol, the approval or permit number that was issued, and the date that approval was granted.

>>> An ethics approval was granted by the Human Research Ethics Committee of the University of Electronic Science and Technology of China (Approval Number: 1061420211102001) in November 2021.

The above information has been added to the Methods section of the manuscript on page 7.

4. Please include a complete copy of PLOS’ questionnaire on inclusivity in global research in your revised manuscript. Our policy for research in this area aims to improve transparency in the reporting of research performed outside of researchers’ own country or community. The policy applies to researchers who have travelled to a different country to conduct research, research with Indigenous populations or their lands, and research on cultural artefacts. The questionnaire can also be requested at the journal’s discretion for any other submissions, even if these conditions are not met.  Please find more information on the policy and a link to download a blank copy of the questionnaire here: https://journals.plos.org/plosone/s/best-practices-in-research-reporting. Please upload a completed version of your questionnaire as Supporting Information when you resubmit your manuscript.

>>> A complete copy of the questionnaire has been provided as supporting information S1.

5. Thank you for stating the following financial disclosure:

This work was supported in part by a grant from the National Natural Science Foundation of China (grant number: 71902017). Please state what role the funders took in the study.  If the funders had no role, please state: "The funders had no role in study design, data collection and analysis, decision to publish, or preparation of the manuscript." If this statement is not correct you must amend it as needed.  Please include this amended Role of Funder statement in your cover letter; we will change the online submission form on your behalf.

>>> The funders had no role in study design, data collection and analysis, decision to publish, or preparation of the manuscript. This has been added to the financial disclosure on P. 17.

6. Please include captions for your Supporting Information files at the end of your manuscript, and update any in-text citations to match accordingly. Please see our Supporting Information

>>> Captions for the Supporting Information files have been provided at the end of the manuscript.

7. In the abstract, the method needs to be better described.

>>> The Method section in the abstract has been enhanced as follows.

*Five key components of an inspection intervention, identified from a focus group discussion, were used as the attributes of a discrete choice experiment (DCE). In the DCE, participants were presented with two hypothetical scenarios and asked to choose the scenario in which they were more willing to comply with the laboratory safety guidelines. Data were collected from 35 clinical laboratories in seven healthcare institutes located in Chengdu, China. In total, 188 laboratory workers completed the DCE. The collected data were analyzed using conditional logit regression and latent class analysis.*

8. Methods need to be improved, especially about attributes and levels and about A and B classes.

>>> The Methods section has been revised to include more details. Firstly, additional information has been provided regarding the definitions of each attribute and their corresponding levels in the Discrete Choice Experiment (DCE). This ensures clarity and facilitates a better understanding of the experimental design.

Moreover, the steps involved in utilizing the latent class model (LCM) to divide respondents into class A and class B have been outlined. These steps include specifying the LCM, estimating its parameters, and assigning respondents to their respective subgroups based on their posterior probabilities of membership.

Please refer to the responses provided for Question 2a by Reviewer 1 and Question 2 by Reviewer 2 for more details.

9. In the results, I believe that other data and results could be incorporated for a better understanding of the presented data of compliance.

>>> In the Results section, we have incorporated the participants' demographic information, including age, gender, education, years of work experience, employment type, and lab size, for further analysis. These results are presented in six tables as part of the Supporting Information. Each table focuses on a specific demographic variable, dividing the respondents into different groups. Each table shows that the coefficients vary in terms of direction and significance among the different groups, indicating the presence of heterogeneity. For more detailed information, please refer to the response to Question 3a from Reviewer 1.

10. The discussion can be broadened, with more foundation in the literature.

>>> Additional discussion regarding the attributes and findings of this study has been included in the Discussion section on page 14, as outlined below.

*To design an appropriate inspection intervention, our study confirmed that the three attributes considered in this study were crucial to the success of an intervention. The first attribute was about the inspector. As suggested by Woodcock (24), a suitable inspector should be experienced and knowledgeable, so as to have a better understanding about the safety regulations and standards. Additionally, a suitable inspector should be characterized by objectivity to provide unbiased and objective evaluations of compliance. From the perspective of the clinical lab workers, our study supported Woodcock’s argument by showing that the person responsible for inspection could affect a worker’s willingness to comply with the safety guidelines. Therefore, selecting a suitable inspector is critical to ensure the effectiveness of inspection intervention.*

*In terms of the inspection frequency, we found that clinical lab workers preferred regular inspections, such as monthly inspections. This might be because regular inspections could translate an organization’s commitment to maintain a safe working environment into perceivable actions, and promote a culture of safety in the workplace (25, 26). Regarding the timing of a safety inspection, although both the fixed and the randomized timings of an inspection had their merits, we found that lab workers preferred random inspections over fixed ones. This finding was consistent with the recommendations made by Berte (27) and Wyllie et al. (28). One possible reason for this preference was that a randomized inspection was more effective in enhancing the lab workers’ safety awareness to motivate them to remain vigilant at all times, thereby reducing the risk of safety incidents. The result that the lab workers preferred regular but randomized safety inspections (e.g., when an inspection occurred on an unspecified day per month) implied that they welcomed random but not too random inspections. This indicates that the workers appreciated a balance between safety practices and smooth business operations.*

*Finally, providing performance feedback was also a critical component in the design of an inspection intervention. Consistent with previous studies (29, 30), our study found that lab workers were more likely to comply with safety guidelines if they were informed about the inspection results. This is reasonable because having performance feedback is helpful to create a sense of accountability, reinforce safe habits and encourage continued engagement in the safety process. In addition to providing performance feedback, consequences based on performance should be considered as well. We found that compared to no consequences, providing appropriate rewards or punishments could encourage lab workers to comply with safety guidelines. If there were a lack of consequences after a safety inspection, a negative safety culture might arise, where safety was not seen as a priority, leading to poor or no safety awareness among individuals.*

**Responses to Reviewer 1**

**Major areas of potential improvement**

1. Introduction:

a. The authors should clearly emphasize their contributions to the existing literature in the introduction rather than in the discussions.

>>> To emphasize our contributions to the existing literature, we wrote the following paragraph in the Introduction on page 3 and page 4.

*Our study contributes to the extant literature in several ways. First, we addressed a significant gap in the safety management of clinical laboratories by identifying evidence-based best practices for inspection interventions. Secondly, we adopted an employee-centered approach, rather than a top-down approach, to gather solid evidence for identifying the most suitable interventions. Specifically, these inspection interventions systematically addressed the clinical lab workers’ preferences to the interventions. Since the ultimate goal of an intervention is to ensure that lab workers are more willing to comply with biosafety guidelines, understanding how clinical lab workers respond to interventions is essential and indispensable when designing effective interventions.*

2. Method:

a. It should be an additional subsection where the authors discuss the attributes and their levels.

>>> The following paragraphs about the definitions of each attribute and their corresponding levels have been added to the Methods section on page 5 and page 6.

*A “lab safety inspector” was defined as a professional, who specialized in evaluating the safety practices of the lab. Having an appropriate safety inspector is crucial in ensuring compliance with relevant safety guidelines to prevent accidents, injuries, and illnesses from occurring. Based on the focus group discussion, four different levels or types of safety inspectors were determined for this attribute. They were the lab director, the group leader, the safety committee member, and the external expert.*

*The attribute “inspection frequency” referred to how often safety inspections were conducted in a clinical lab. A safety inspection could be conducted regularly or not. A regular safety inspection may help to identify safety hazards and unsafe practices in a timely manner and could provide a consistent level of safety oversight. Based on the discussion of the focus group, four levels were assigned to describe this attribute. They were a weekly inspection, a monthly inspection, an inspection only before an audit, or an inspection after an audit.*

*The attribute “inspection timing” indicated whether each safety inspection would be scheduled at a fixed (pre-scheduled) or a random time. Unlike a pre-scheduled safety inspection, a randomized inspection would be unpredictable and may help inspectors to see the real safety condition of a lab, but the random occurrence of the inspection may disrupt their operations and undermine a lab’s capability for planning.*

*The attribute “communication of outcome” referred to how a clinical lab provided its workers with feedback about the results of the safety inspections. Feedback, if delivered properly, helps workers to improve their performance. There were various ways of delivering feedback. In this study we considered four feedback delivery options, following the discussion of the focus group. They were either no feedback provided, sending an email to each inspected lab worker, verbally communicating the results with lab workers through their supervisors, or publicly posting the inspection outcomes.*

*The last attribute “reward/punishment” indicated the consequences of safety inspections. The consequence could either be a reward or a punishment given to lab workers based on their adherence to safety guidelines. Based on the focus group discussion, four levels or consequence options were identified. They were no consequence at all, having a discussion with a supervisor, receiving retraining, and receiving recognition.*

b. The authors should explain in more detail how the baseline scenario was defined. Since the authors mentioned that “To achieve this goal, the baseline case as the scenario in which all the attributes were set to their reference levels (see Table 1) was defined”. So, did the author mention that the baseline/status quo option is where all attributes were at the reference level? However, I observed that in the questionnaire (Supplementary material) the authors said that there were only two alternative scenarios A and B that the respondents can choose. Is there no status quo option in each choice question that respondents can choose in case they prefer neither A nor B?

>>> In this paper, the baseline does not represent the status quo. In a DCE, there is no need to establish a status quo, as participants are given hypothetical scenarios to make their trade-off decisions among different attribute levels. We used the strategy to pick one fundamental level of an attribute as its reference level, which is a common practice in conducting DCEs.

Furthermore, in the discrete choice experiment with binary options (e.g., A or B), where the pairwise comparison is easy to interpret, either A is preferred to B, or vice versa. Even in a situation when the participants preferred neither A nor B, the participants were still asked to select one option, A or B, with which they were more willing to comply. This requirement was clearly mentioned in the guidelines of the questionnaire and was emphasized in the verbal introduction to the participants prior to the survey. Theoretically, the inclusion of this mandatory choice has several advantages. First, it promotes active engagement from the respondents, encouraging them to carefully consider their choices and provide more comprehensive information about their preferences. Secondly, the mandatory choice contributes to noise reduction in the data. Lastly, the mandatory choice facilitates a better understanding of the trade-offs the participants are willing to make. Overall, the inclusion of a mandatory choice enhances the quality and reliability of the data, leading to a more robust analysis of the respondents' preferences.

c. Following this, how the baseline of the dependent variable in the Probability model can be defined? Is Scenario A defined as a baseline? The authors should carefully verify this issue; otherwise, the results are problematic.

>>> As we have clarified that scenario A was not considered as the baseline case in this study in the previous question 2b, here we answer how the probability model was constructed. This explanation has been provided in Supporting Information S2.

*In our discrete choice experiment, the explainable utility* $V_{ij}$ *for clinical lab worker* $i$ *to choose alternative* $j$ *is*

$V_{ij}=\beta_{1}{GROUP}_{ij}+\beta_{2}{SAFETY}_{ij}+\beta_{3}{EXTERNAL}_{ij}+\beta_{4}{MONTHLY}_{ij}+\beta_{5}{BEFORE}_{ij}+\beta_{6}{AFTER}_{ij}+\beta_{7}{RANDOM}_{ij}+\beta_{8}{EMAIL}_{ij}+\beta_{9}{SUPERVISOR}_{ij}+\beta_{10}{POST}_{ij}+\beta_{11}{DISCUSSION}_{ij}+\beta_{12}{RETRAIN}_{ij}+\beta_{13}{RECOGNITION}_{ij}$	*(S1)*

*In a conditional logit model, individuals are assumed to be homogeneous. Therefore, the coefficient vector* $\boldsymbol{\beta}$ *in Equation (S1)* *is independent of the respondents, and the index* $i$ *in Equation (S1) can be dropped. In general, the probability of choosing alternative* $j$ *over alternative* $k$ *can be calculated as*

$P_{j}={e^{V_{j}}}/{(e^{V_{j}}+e^{V_{k}})}$	*(S2)*

*Since the choice in our study is binary (yes or no), Equation (S2) for calculating a probability can be further simplified as*

$P_{yes}={e^{V_{yes}}}/{(1+e^{V_{yes}})}$	*(S3)*

*Because effects coding was used in our study, the number of new variables created in effects coding was equivalent to the number of levels of the attribute being coded, minus one. For instance, there were four levels in the attribute of lab safety inspector. We created three new variables, such as GROUP, SAFETY, and EXTERNAL.*

*Since the level “lab director” was defined as the reference level, setting GROUP, SAFETY, and EXTERNAL to be -1 could represent lab director. In the baseline case, all attributes were set to their reference levels, and the coding for these reference levels was presented in Table 1.*

*Next, we use the baseline intervention as an example to calculate its corresponding probability of compliance for this intervention. Note that in the baseline intervention, all attributes were set to their reference levels, and the coding for these reference levels was presented in Table 1. For example, for the attribute of lab safety inspector, “lab director” was defined as the reference level. Therefore, we coded GROUP, SAFETY, and EXTERNAL to be -1 to represent the lab director.*

*Using the coefficients of the regression results shown in Table 3, the explainable utility* $V$ *at the baseline intervention is equal to*

$$V=-0.0671\times(-1)+0.2155\times(-1)-0.1205\times(-1)+0.2493\times(-1)-0.0087\times(-1)-0.3451\times(-1)+0.0766\times(-1)+0.1776\times(-1)+0.0914\times(-1)+0.0618\times(-1)+0.0435\times(-1)+0.3558\times(-1)+0.3231\times(-1)=-1.0532$$

*Furthermore, plugging* $V=-1.0532$ *into* $V_{yes}$ *in Equation (S3) yields the probability of the baseline intervention as 25.86%.*

3. Results and discussions:

a. Does Table 3 present the results without controlling for individuals’ demographic characteristics? If yes, it will be beneficial to have an estimation result with the control variables.

>>> Yes. The results presented in Table 3 did not control the individuals’ demographics. But we have now provided additional analyses by incorporating the participants’ demographic information as control variables. The additional results are presented in Tables S3 – S8. Using Table S3 as an example, which presented the regression results with respect to the respondents' age, the coefficients for the three age groups differed in terms of both direction and significance, implying the presence of heterogeneity across the three different age groups. Heterogeneity can also be observed in the other five tables S4– S8 included in the appendix. Overall, in these six tables in the appendix, only one single demographic characteristic was considered each time. To further understand how heterogeneity affected the results across multiple demographics, rather than just one characteristic, a more sophisticated method based on latent class analysis was employed in the paper.

The above information has been provided in the section of Results on page 10.

b. It is better to specify the difference between Classes A and B. For instance, can we say that respondents in Class B are more “reward/punishment-sensitive” than those in Class A? and that Class A is a “compliant group”?

>>> Thank you for the comment, which is well taken. It’s true that the class B laboratory workers would be more willing to comply with safety guidelines if there were punishments or rewards after the inspection. They were indeed more reward/punishment-sensitive and we described them as more outcome-oriented in the paper. As for class A workers, indeed they would have a high compliance rate of 85.48% under the best practice inspection intervention. But class B could achieve a high compliance rate as well at 81.4% under the corresponding best practice. As a result, we do not label class A as a “compliant group”, but a more “process-oriented” group, as the workers were more concerned about the process of the inspection, such as who conducted the inspection and the frequency of inspection (from Table 5).

We have elaborated the difference between classes A and B, and have added a new paragraph in the Results section on page 12, which is also documented below.

*According to the above discussion, clinical lab workers in class A could be described as more “process-oriented”. This is because their preferences were determined mainly by how the inspection was conducted (e.g., the inspector and the frequency of the inspection). On the other hand, class B lab workers were more “outcome-oriented”, as their compliance strongly depended on the consequence of the inspection (e.g., reward or punishment).*

c. The authors argued that class A laboratory workers preferred for safety inspections to be conducted by an external expert. However, the Results of Table 5 suggest only a negative and significant impact of “By a group leader” at a 10% level. So, they should be indifferent to “Lab safety inspectors” at a 5% significant level.

>>> We agree with the reviewer’s observation that class A lab workers did not display any significant preference for the type of inspector at the 5% significant level. On the other hand, the probability model that makes use of the regression results to project the probability of compliance under each hypothetical setting is valid, as it is a well-established method. It’s also true that the obtained best practice inspection intervention involved using an external expert as the inspector. Therefore, we made the following changes in the manuscript to address your concern: (i) we stated that the best practice inspection interventions were identified to yield the highest projected compliance with the safety guidelines; and (ii) the best practice intervention for class A laboratory workers “involved” using an external expert as the inspector. We no longer mention that class A workers “preferred” using an external expert as the inspector. We hope that you will agree with our revision.

**Minor areas of potential improvement**

1. The authors should explain what is meaning of the coding matrix (-1,1,0) in Table 1.

>>> Table 1 has been revised to clarify the meaning of the coding matrix. An additional paragraph has been added in the Methods section on page 6 for clarification.

*In our study, we utilized effects coding. This approach involved creating a number of new variables, which was the number of the levels of an attribute being coded, minus one. For instance, the attribute of lab safety inspector had four levels, so we created three new variables, namely GROUP, SAFETY, and EXTERNAL. Since the level “lab director” was defined as the reference level, we set the values of GROUP, SAFETY, and EXTERNAL for “lab director” to be -1, as shown in Table 1.*

2. The first paragraph of the Strengths of the paper is better to be discussed in the introduction.

**>>>** As suggested by the reviewer, the first paragraph of the Strengths of the paper has been moved to the introduction.

**Responses to Reviewer 2**

1. Please spelled out the full name, such as HCW (Line 86), SE (Table 3-5).

>>> The full name with abbreviation has been provided. For instance, HCW stands for health care worker, and SE is an abbreviation for standard error.

2. About Class A and Class B: Please describe in detail Class A and Class B, including how to divide the Class A and Class B in Method section, the characteristics of Class A and Class B. It is necessary to make these clear for reader.

>>> The following paragraph, which explains how to divide Class A and Class B, has been improved in the Method section on page 7 and page 8.

*The data were analyzed by Stata V.15.1 for Windows. A conditional logit model was used to investigate the impact of the key attributes of an inspection intervention on the laboratory workers’ compliance with safety guidelines. In addition, a latent class model (LCM) was used to evaluate the heterogeneity among the respondents. The LCM divided the respondents into several subgroups based on their responses to the choice sets (22). The appropriate number of subgroups was determined by assessing a set of performance indicators, such as the Akaike Information Criterion (AIC), the Constrained Akaike Information Criterion (CAIC), and the Bayesian Information Criterion (BIC). The number of segments with the minimum values of performance indicators was preferred (23). After the number of subgroups was determined, based on the posterior probabilities of membership in each subgroup, each respondent was assigned to the subgroup with the highest probability of membership. In other words, the LCM divided the respondents into subgroups based on their similar response patterns while also maximizing the differences between these subgroups.*

3. Results description is not very clear. For example, the baseline case, and the calculation the percentage (Line 189-191, Line 235-236, Line 240-242, Line 250-254, Line 269-270…).

>>> The explanations for the baseline case (Line 189-191 in the previous version) and the calculation of the probability of a hypothetical intervention scenario (Line 235-236, Line 240-242, Line 250-254, Line 269-270 in the previous version) have been addressed in the revision. We have added a paragraph at the end of the data analysis section on page 8 to briefly describe how the Discrete Choice Experiment (DCE) methodology can be used to project the compliance (or probability) of a hypothetical intervention scenario. We have also provided how the probability of a scenario is calculated with an example in the Supporting Information S2. On page 10, we now describe how we evaluated all 512 possible intervention scenarios to identify the best practice, which is applied to both the homogeneous and classified cases.

4. The English is not of sufficient quality for an international peer-reviewed journal.

>>> The revision has been reviewed and edited by a professional editor, who is a native English speaker.
