# Supplementary material for: Best practices for implementing biosafety inspections in a clinical laboratory: Evidence from a multi-site experimental study
Source: PLoS One. 2023 Oct 13;18(10):e0292940. doi: 10.1371/journal.pone.0292940 (PMC10575490; doi:10.1371/journal.pone.0292940)
Supplement: Attachment — Submitted filename: response letter.docx [file pone.0292940.s013.docx]

**Responses to the Reviewers’ Comments**

The authors would like to thank the review team for their thoughtful, thorough, and constructive feedback on our previous submission. We appreciate the opportunity to further strengthen our research.

**Responses to the Academic editor**

1. Please review your reference list to ensure that it is complete and correct. If you have cited papers that have been retracted, please include the rationale for doing so in the manuscript text, or remove these references and replace them with relevant current references. Any changes to the reference list should be mentioned in the rebuttal letter that accompanies your revised manuscript. If you need to cite a retracted article, indicate the article’s retracted status in the References list and also include a citation and full reference for the retraction notice.

>>> Thank you for the feedback. We have carefully reviewed our reference list and ensured that it is complete and correct. We can confirm that none of the cited papers in our manuscript have been retracted and all references are relevant and current.

2. Please ensure the clear and comprehensive explanation of data availability in the content and provide a detailed justification on the appropriate statistical analysis employed, demonstrating its correctness and validity.

>>> Thank you for your feedback. We have addressed the issues of data availability and the appropriate statistical analysis in the revised manuscript. Regarding data availability, we have now provided all data, including the survey data in Supporting Information S2, and detailed evaluation of all 512 scenarios for determining best practices in Supporting Information S3A and S3B.

**Responses to Reviewer 2**

**Major areas of potential improvement**

1. This study provides a valuable approach for designing best practice inspection interventions, and these interventions would play an important role for biosafety management of clinical laboratories and biosafety laboratories. It would improve the compliance with biosafety guidelines through identifying evidence-based suitable interventions, rather than current top-down approach mainly used. There is a need to describe the detailed methods and related results for this approach in the manuscript for wider application in actual life.

>>> Thanks for your comments. In this version, we have made improvements in describing the methods and related results. More specifically, we improved the descriptions of the LCM (Lines 197-209) and the heterogeneous responses of Classes A and B (Lines 273-274). We also illustrate how customized interventions may be implemented based on our results (Lines 443-456).

**Minor areas of potential improvement**

1. Line 48: “to aid physicians in in the diagnosis…”, please delete the extra “in”.

>>> Thank you for your suggestion. The extra “in” has been removed from the sentence.

2. About Class A and Class B: The description for them remains unclear. It may be easier to understand in Method section, such as “In other words, the LCM divided the respondents into subgroups, such as Class A and Class B, based on their similar response patterns while also maximizing the differences between these subgroups.” (Line 224-226). Or these subgroups can be tried to explain in Results section: According to the LCM, two subgroups, Class A and B, were identified based on their similar characteristics related to their safety guidelines compliance intentions, where …… (Line 295-297). Please consider seriously and make an appropriate revision.

>>> Thank you for your feedback. We have made the necessary revisions in accordance with your suggestion. Specifically, in the Method section (Lines 208-209), we have added an additional explanation of Class A and Class B as subgroups identified through Latent Class Modeling (LCM) as follows:

*In other words, the LCM divided the respondents into subgroups, based on their similar response patterns while also maximizing the differences between these subgroups.*

Additionally, in the Results section (Lines 273-275), we have further clarified the characteristics of Class A and Class B in relation to their safety guidelines compliance intentions as follows:

*Using the LCM, two subgroups (Class A and Class B) of the respondents with distinct responses to safety guidelines were identified, where Class A represented 31.91% of the cohort, and Class B was the remaining 62.09%*

3. Some important results could be described more clearly like S2, such as “For class A, this inspection configuration could yield compliance at 85.48%. (Line 343-344)”,“With this intervention configuration, class B compliance could be as high as 81.40%. (Line 350-351)”, Line 360-364, Line 414-415, Line 281-283 etc. After all, there is a need to explain clearly to a wider audience how these data were obtained, and it may be better to present these results in a table rather than just words.

>>> Thank you for your valuable feedback. To provide a clearer understanding for a wider audience, we have provided a detailed explanation of how each probability was calculated in Supporting Information S3A using an example. Furthermore, in Supporting Information S3B, we provide a spreadsheet that contains the calculation of the probabilities of all 512 scenarios for the homogeneous, Class A, and Class B cases. See Lines 253-256:

*We show in detail how the probability analysis was conducted using the baseline as an example in Supporting Information S3A. In Supporting Information S3B, we provide a spreadsheet showing how the probabilities of all 512 intervention scenarios were calculated.*

To help the reader to understand how the probability of a mentioned scenario is calculated, we refer to the intervention scenario number in S3B for the reader to check. The following is one example in Lines 262-264.

*The projected compliance of this best practice intervention could improve the probability of compliance from 25.86% at the baseline case (Homogeneous Scenario # 186 in S3B) to 74.54% (Homogeneous Scenario # 488 in S3B).*

4. The author mentions the “baseline” several times in the manuscript, but it remains unclear what it is. It is necessary to explain it or give an appropriate description.

>>> Thank you for your feedback. The baseline case is now defined clearly. Please see a new paragraph in Lines 216-220 in the manuscript, which is shown below.

*In DCEs, each scenario was analyzed against a baseline case, which referred to the scenario where all attributes were set to their reference levels as defined in Table 1. This includes the laboratory safety inspector being the lab director, a weekly inspection frequency with a fixed day and time, no feedback on an inspection outcome, and no consequences irrespective of the inspection outcome.*
